# Synthesis of Hybrid Organic-Inorganic Hydrotalcite-Like Materials Intercalated with Duplex Herbicides: The Characterization and Simultaneous Release Properties

**DOI:** 10.3390/molecules26165086

**Published:** 2021-08-22

**Authors:** Sheikh Ahmad Izaddin Sheikh Mohd Ghazali, Is Fatimah, Farah Liyana Bohari

**Affiliations:** 1 Faculty of Applied Sciences, Universiti Teknologi MARA Cawangan Negeri Sembilan, Kampus Kuala Pilah, Kuala Pilah 72000, Negeri Sembilan, Malaysia; liyanabohari@gmail.com; 2 Department of Chemistry, Faculty of Mathematics and Natural Sciences, Universitas Islam Indonesia, Kampus Terpadu UII, Jl. Kaliurang Km 14, Sleman, Yogyakarta 55584, Indonesia; isfatimah@uii.ac.id

**Keywords:** 2,4,5-trichlorophenoxybutyric acid (TBA), 3,4-dichlorophenoxy-acetic acid (3,4D), controlled-release, herbicides, layered double hydroxide (LDH), nanohybrid composites

## Abstract

In this study, a controlled-release formulation of duplex herbicides, namely, 2,4,5-trichlorophenoxybutyric acid (TBA) and 3,4-dichlorophenoxy-acetic acid (3,4D), was simultaneously embedded into Zn-Al-layered double hydroxides (LDHs). The resulting nanohybrid Zinc-Aluminium-3,4D-TBA (ZADTX) was composed of a well-ordered crystalline layered structure with increasing basal spacing from 8.9 Å to 20.0 Å in the Powder X-ray Diffraction (PXRD) with 3,4D and TBA anions located in the gallery of LDHs with bilayer arrangement. The release of 3,4D and TBA fit the pseudo-second-order model. This duplex nanohybrid possessed a well-controlled release property (53.4% release from TBA and 27.8% release from 3,4D), which was highly effective, requiring the use of a small quantity and, hence, environmentally safer.

## 1. Introduction

The layered double hydroxide (LDH) as hosts for agrochemicals, pesticides, and herbicides has long been used to reduce the number of active chemicals used in agriculture [1]. Chemically, LDH and layered hydroxide salts are compounds derived from brucite (Mg(OH)_2_). The LDH is formed when some of the divalent cations are replaced by trivalent cations, resulting in excess of positive charges that are balanced by the intercalated anions. The LDH is represented by the general formula [$M_{1-x}^{2+}M_{x}^{3+}$(OH)_2_]^x+^(A^n−^)_x/_n.yH_2_O, in which M^2+^ and M^3+^ are divalent and trivalent metallic cations, whereas A is a hydrated counter ion with charge n^−^ [2,3]. Various LDH hosts have been employed in the production of controlled-release formulations using encapsulation techniques to avoid evaporation, oxidation leaching, and the improper use of hazardous chemicals [4,5,6]. In general, the interlayer area of lamellar host LDHs functions as a microvessel to store active molecules, such as drugs [7], herbicides [8], pesticides [9], and plant growth regulators [10] for forming organic-inorganic nanohybrids with controlled-release property. In agriculture, agrochemical herbicides such as 3,4-dichlorophenoxy-acetic acid(3,4D) and 2,4,5-trichlorophenoxybutyratic acid (TBA) are used to kill weeds in paddies and cornfields [11,12,13] in massive quantities. When these agrochemical residues were washed away by rain, the run-off and leaching from the soil would contaminate waterways [14].

In this study, a new layered organic-inorganic nanohybrid was synthesised by intercalating two anions, 3,4D and TBA, simultaneously into LDH interlayers. The simultaneous controlled release of the resulting intercalated phenoxy herbicides from the nanohybrid into the aqueous solution of phosphate anions was also studied. Given that the preparation and simultaneous controlled release of both phenoxy herbicides anions from LDH interlayer are available in the literature, the physico-chemical properties of the synthesised nanohybrid containing two active agents that might affect the release were also evaluated. The findings of this study would alleviate the problems of agrochemicals contaminating waterways, via the use of LDHs as packaging, transport, and distribution materials for herbicides, by minimising the number of chemicals required for agriculture.

## 2. Results and Discussions

### 2.1. The PXRD Analysis

**Figure 1:**
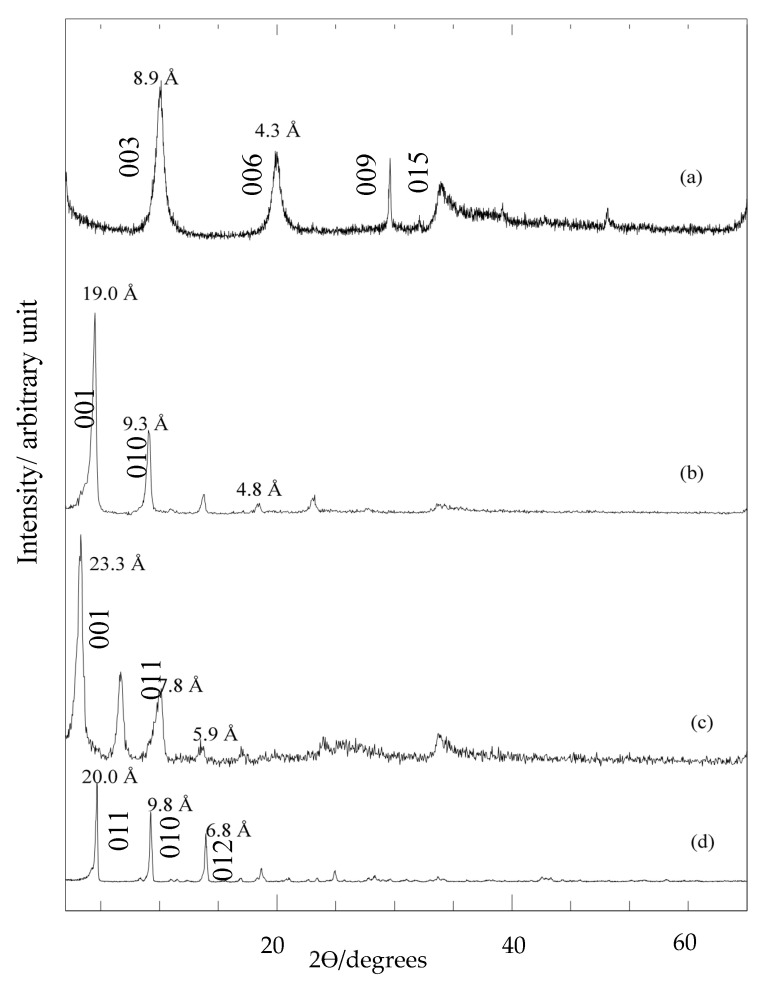
PXRD patterns of (**a**) LDH, (**b**) nanohybrid intercalation with single anion, 3,4D (ZAMDX) and (**c**) TBA (ZATX) and (**d**) nanohybrid containing both anions, 3,4D and TBA (ZADTX).

Figure 1 shows the PXRD patterns and basal spacing for the LDH nanohybrid intercalated with dual herbicides, 3,4D and TBA, in which the basal spacing of ZADX (Figure 1b) and ZATX (Figure 1c) was 19.0 Å and 23.3 Å, respectively. Both samples showed a high intensity and sharp reflection, indicating good crystallinity and well-ordered nanolayered materials. Compared to LDH, the basal spacing for both ZADX and ZATX expanded by 8.9 Å (Figure 1a), confirming the inclusion of 3,4D and TBA, respectively. Also, compared to the 2Ɵ of 9.8° for LDH, ZADTX showed sharp and symmetric peaks at a lower 2Ɵ angle of 4.6° (Figure 1d), indicating that the hydroxide layers were further expanded upon the intercalation of bulky anionic molecules [15]. The PXRD patterns of ZADTX prepared at 0.1 M of TBA and 3,4D anions gave rise to a new monophasic nanocomposite through dual intercalation. This new nanocomposite showed a slightly larger basal spacing value (20.0 Å) compared to ZADX (19.0 Å) but slightly smaller to ZATX (23.3 Å). Factors such as anionic size, charge, orientation, and interaction with the positively charged inorganic interlayer affect the degree of intercalation and the separation between layers. Zn/Al-NO3–LDH showed basal spacing of 8.9 Å, a typical basal spacing shown by Zn/Al-NO3–LDH with nitrates as the counter anion. Increment in basal spacing in herbicides/LDH nanocomposites is due to displacement of nitrates with larger organic herbicide molecules.

**Figure 2:**
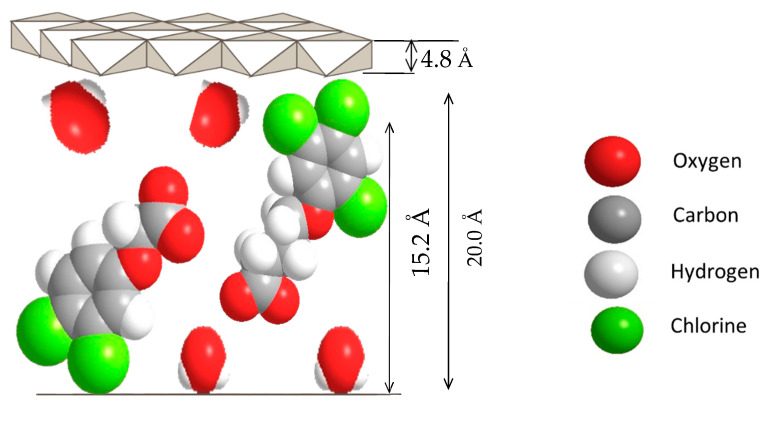
Proposed orientation of 3,4D and TBA in LDH interlayer for the formation of dual-guest nanocomposite ZADTX.

Figure 2 shows the proposed arrangement of ZADTX within the LDH interlayer region, in which the gallery height was calculated to be 16.2 Å upon subtracting the thickness of the brucite layer (4.8 Å) based on the PXRD basal spacing d003 of 20.0 Å for ZADTX, and its tilting angle was calculated as 49° for 3,4D and 34.6° for TBA. The TBA might have a higher affinity towards the LDH interlayer, probably because it had three chlorine atoms for electrostatic bonding compared to 3,4D, which had only two chlorine atoms. This additional chlorine atom enabled TBA to be more easily intercalated and held stronger within the positively charged layers than the 3,4D anion. The empirical formula of Zn/Al-LDH nanocomposites and ZADTX nanomaterial are listed in Table 1, was determined from ICP-AES and TGA/DTG analyses.

### 2.2. The UV-Visible Spectrometry and Direct Injection Mass Spectra (DIMS)

**Figure 3:**
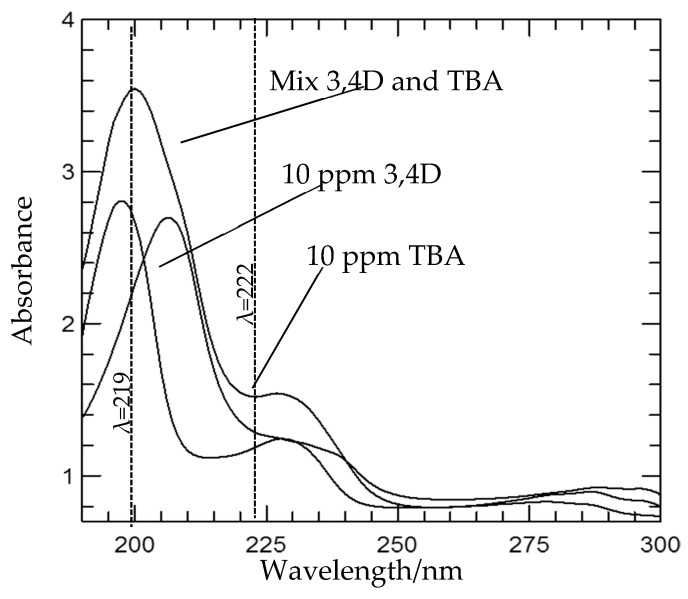
UV-Vis spectra for 3,4D and TBA with λmax at 219 and 222 nm.

Figure 3 shows the loading percentage of TBA and 3,4D was found to be 23% and 10% (*w*/*w*), respectively. The uptake of TBA was higher than 3,4D anion, probably due to its stronger affinity to the high-charged density of the LDH interlayers, in which three chlorine atoms were bonded with Zn^2+^ ions in the former compared to only two in the latter [16].

**Figure 4:**
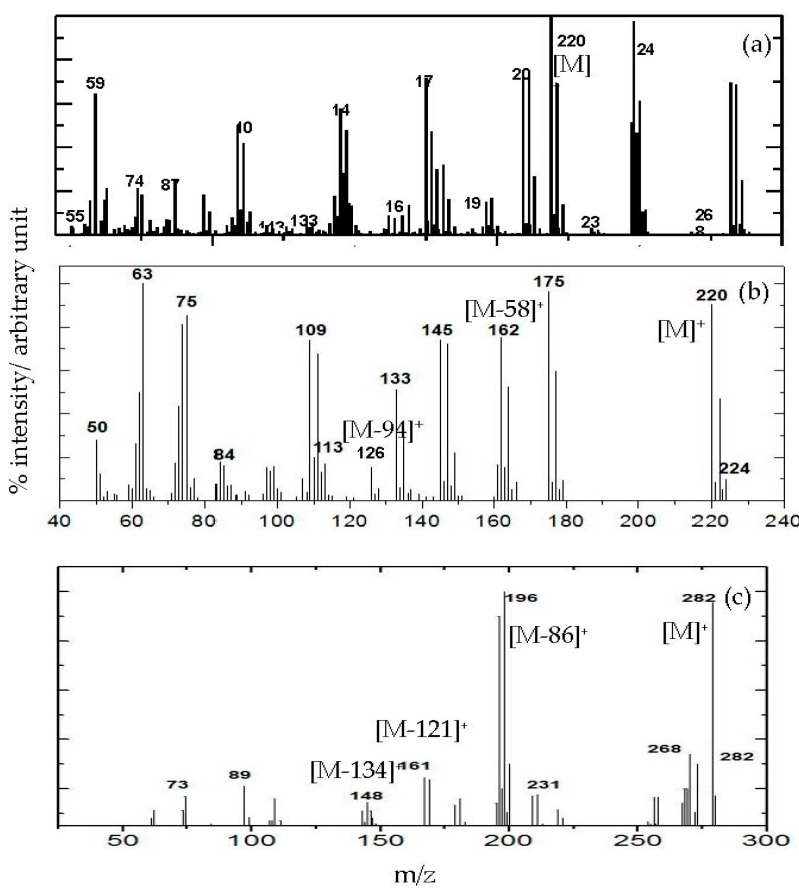
DIMS pattern of dual guest nanocomposite (**a**) TBA, (**b**) 3,4D and (**c**) ZATDX.

To confirm the intercalation of 3,4D and TBA into the LDH interlamellar, the DIMS analysis of 3,4D and TBA was compared to that of ZADTX-LDH. Figure 4 gives the DIMS of TBA, 3,4D, and dual guest nanocomposites of ZADTX Mass spectra of pure TBA with an intense peak at *m*/*z* 220 for pure TBA (Figure 4a), while pure 3,4D (Figure 4b) showed an abundant peak at *m/z* 282. These peaks could be ascribed to the molar mass of the respective compound. The mass spectroscopy of TBA displayed the parent ion at *m/z* = 282 and characterised peaks at *m*/*z* = 196, 161, and 148. The spectrum of TBA anion also gave an intense peak for the C_7_H_6_Cl_3_O^+^ phenoxy group, with an *m*/*z* value of 196, probably due to the loss of the C_3_H_6_CO_2_^+^ ion. The loss of ion Cl^−^ gave a peak at *m*/*z* = 161. On the other hand, the peak at *m*/*z* = 148 was probably due to the fragmentation of CH_2_^+^ ion to form the C_6_H_5_Cl_2_O^+^ phenoxy ion. The comparatively intense fragmentations of ion [C_6_H_2_Cl_2_O]^−^ (*m*/*z* 162) and [C_6_H_2_ClO]^−^ (*m*/*z* 126) were derived from the dicholorinated phenolate ions of the 3,4D. The high intensity at *m*/*z* 77 corresponded to the deprotonated benzene, which could be attributable to the fragmentation of 3,4D [17]. The presence of *m*/*z* = 282 peak of TBA anions and the *m*/*z* = 220 peak for the 3,4D anion indicated that both anions were simultaneously intercalated into the host interlayer to form the nanohybrid (Figure 4c).

### 2.3. FTIR Spectra

**Figure 5:**
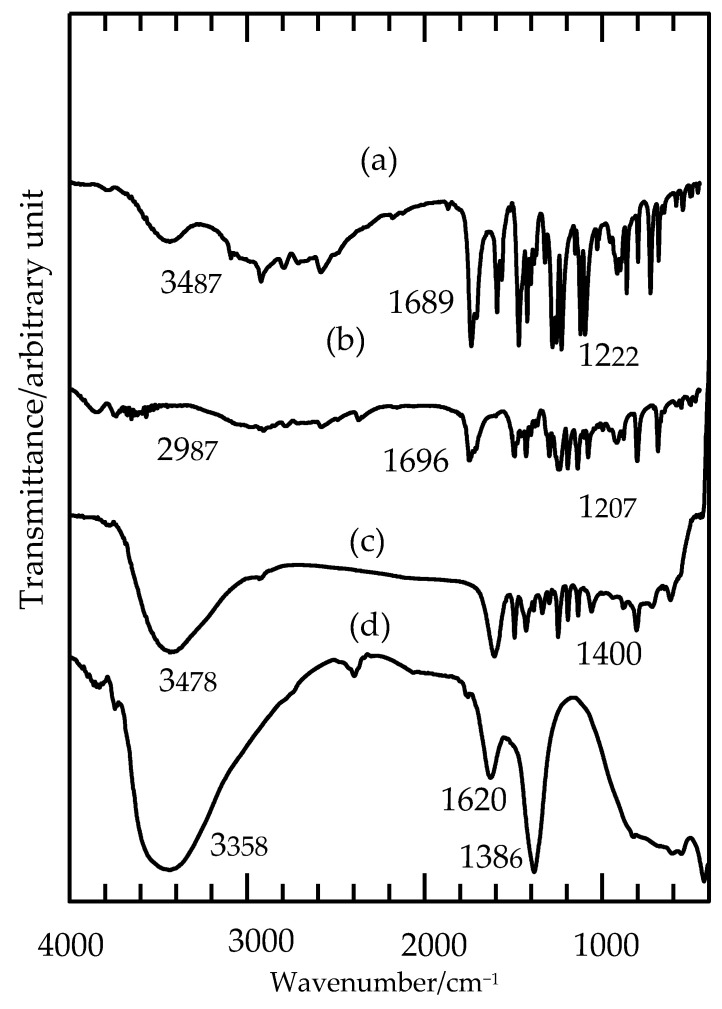
FTIR spectra of the guest anions (**a**) 3,4D, (**b**) TBA, (**c**) ZADTX and (**d**) LDH.

In the FTIR analysis, all nanohybrids showed similar absorption bands because the same functional groups of the 3,4D and TBA were intercalated into the host interlayer galleries. However, some absorption bands were slightly shifted due to the interaction of both anions with the host layer. The typical broad absorption bands of LDH were detected at 3358 cm^−1^ and 1620 cm^−1^, corresponding to the vibration of hydroxyl groups at the surface and the interlayer water molecules in the water-bending mode, respectively [18,19,20]. Figure 5 shows FTIR spectra of the pure anions, in which the band at approximately 2900 cm^−1^ for both anions was probably due to the O-H stretching vibration of COOH, while the strong band at 1700 cm^−1^ corresponded to the stretching of C=O. The bands at 1471 cm^−1^ for 3,4D (Figure 5a) and 1467 cm^−1^ and 1403 cm^−1^ for TBA (Figure 5b) were attributable to C=C vibrations of the aromatic ring. Doublet bands at about 1200 cm^−1^ for both anions were probably due to C–O–C symmetric and asymmetric stretching modes.

On the other hand, the nanohybrid, ZADTX (Figure 5c) showed a combination spectrum of the host LDH and the guest anions, namely, 3,4D and TBA. The bands located at 1467 cm^−1^ and 1409 cm^−1^ were probably due to the stretching vibration of the aromatic ring C=C. The disappearance of bands at 1345 cm^−1^ and around 1700 cm^−1^ in the nanohybrid spectrum were probably due to the vibrational absorption of NO^−^ in the interlayer and the C=O stretching vibration of the protonated carboxylic groups of the herbicides, respectively [21]. The disappearance of these bands showed that the nitrate anions were completely exchanged with TBA and 3,4D anions to form the ZADTX nanohybrid.

### 2.4. The Thermal Analysis

**Figure 6:**
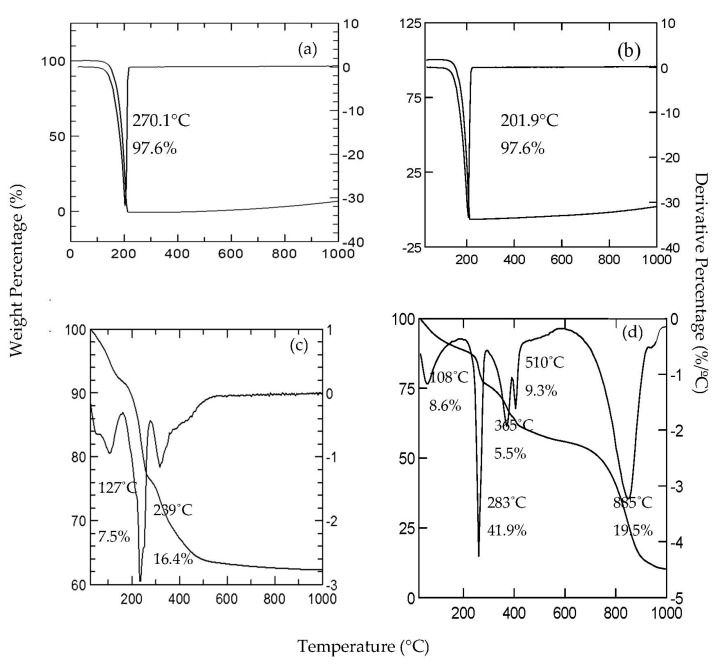
TGA/DTG analyses of (**a**) 3,4D, (**b**) TBA, (**c**) LDH and (**d**) ZADTX.

Figure 6 shows the thermal profiles of decompositions for ZADTX, LDH, 3,4D and TBA, the maximum temperature of 3,4D, and TBA anions (Figure 6a,b) were 270.1 °C and 207.8 °C, respectively. Compared to the decomposition of pure anions 3,4D and TBA, the ZADTX nanohybrid showed a higher thermal temperature, indicating that the intercalated anions were thermally more stable than their non-intercalated forms.

In general, the thermal decomposition of LDHs occurred via three steps: (i) the removal of the physically adsorbed (physisorbed) water at the surface and between the hydroxide layers from room temperature up to 200 °C, (ii) the simultaneous dehydroxylation and decarbonisation of hydroxide layers between 300 and 400 °C, and (iii) the elimination and combustion of the organic anion [22,23]. The weight loss of LDH (Figure 6d) at 100–300 °C corresponded to the elimination of the interlayer structural water. Conversely, the thermal progression of the ZADTX nanohybrid (Figure 6c) took place in five consecutive stages with a total weight loss of 8.6% in the range of 70–300 °C, and all the loss occurred at the first stage up to 108 °C as a result of the water being adsorbed onto (or intercalated into) the ZADTX.

### 2.5. The Surface Property

**Figure 7:**
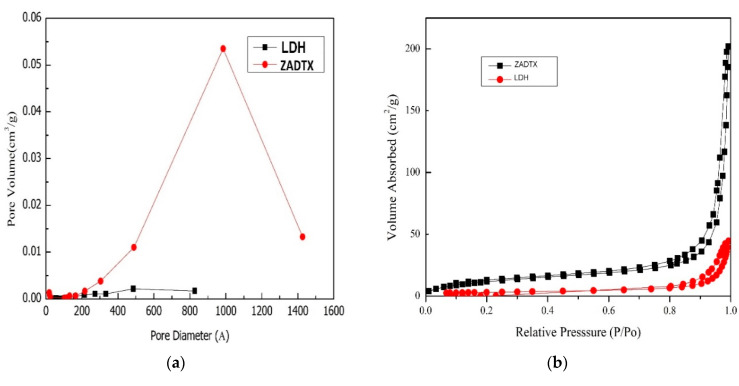
(**a**) Pore size distribution of LDH and ZADTX and (**b**) Nitrogen adsorption-desorption isotherms.

Figure 7 gives the adsorption-desorption isotherms of nitrogen gas on LDH and ZADTX for the ZADTX, LDH host, and nanocomposite. All nanocomposites and the LDH host showed the Type IV isotherm with H3 hysteresis loop of the International Union of Pure and Applied Chemistry (IUPAC) standard, indicating that the materials were mesoporous. This type of H3 hysteresis loop was due to the non-rigid aggregates of plate-like particles such as clay compound [24,25] giving rise to the slit-like pores [26,27]. Overall, the LDH host showed a slow adsorbate uptake of nitrogen at pore volumes 0.0–0.8 cm^3^/g with a maximum adsorbate uptake of 50 cm^3^/g (Figure 7a). However, for the intercalated compound, a rapid adsorbate uptake of nitrogen occurred at pore volumes around 0.0–0.6 cm^3^/g with a maximum adsorbate uptake of 200 cm^3^/g.

Besides, the nanocomposite ZADTX showed a broad distribution of pores with a maximum diameter close to 1000 Å, as shown in the graph of the pore size distribution for the nanocomposite and LDH host (Figure 7b). Table 2 shows the data on BET surface area, BET average diameter, and BJH average diameter, in which the BET surface area of ZADTX was 2.75 m^2^/g, and it was substantially larger than that of the LDH host (1.12 m^2^/g). On the other hand, the BET average diameter for the LDH host and ZADTX were 224.00 Å and 373.02 Å, respectively, while the BJH average diameter of LDH host and ZADTX were 124.00 Å and 588.92 Å, respectively.

Substantial changes in the surface area in the nanocomposite were probably due to the exposure of active sites for the adsorption of both anions 3,4D and TBA to be intercalated in between the interlayer spaces [28,29,30]. These changes also indicated that the LDH intergallery was expandable without damaging the original LDH structure with an increment of the PXRD basal spacing from 8.9 Å in the LDH host to 20.0 Å in the ZADTX nanocomposite [31].

### 2.6. The Release Profile of ZADTX into the Aqueous Solution

**Figure 8:**
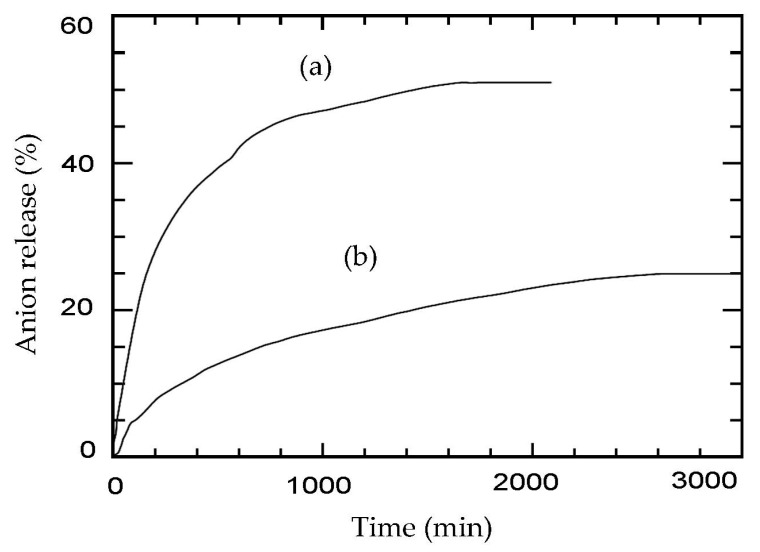
Simultaneous release profiles of (a) TBA and (b) 3,4D from ZADTX into 0.0005 M Na_3_PO_4_ aqueous solutions.

**Figure 9:**
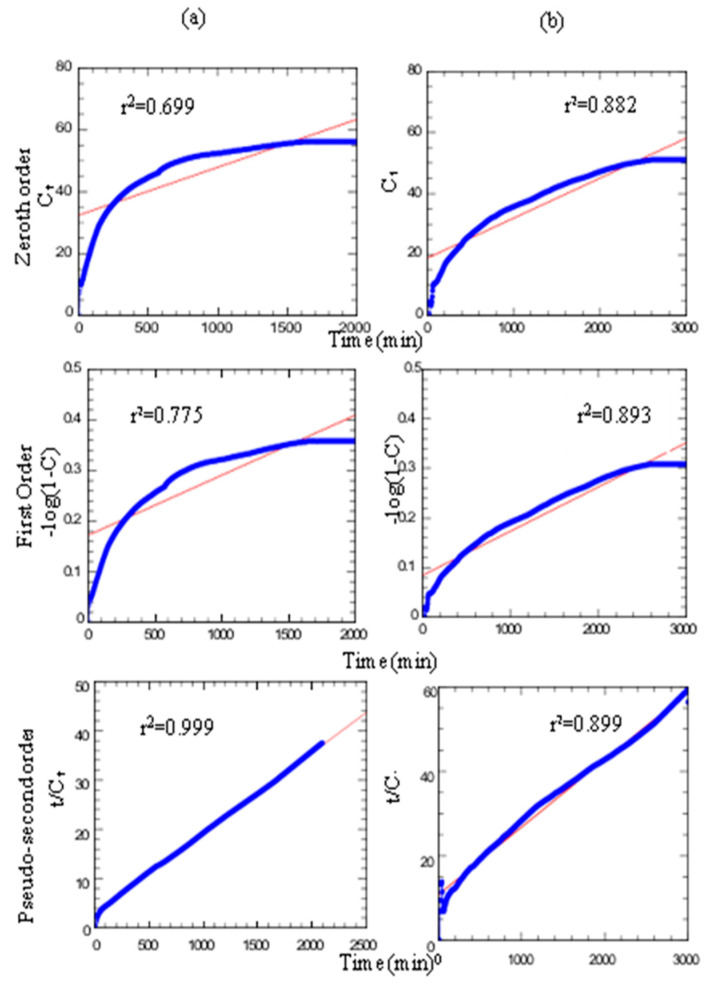
Fitting the release of 3,4D (**a**) and TBA (**b**) from ZADTX nanocomposite to the zeroth- order, first-order, pseudo-second-order and parabolic diffusion kinetics models.

Figure 8 shows the profile of herbicides being released from the ZADTX nanohybrid composite into 0.0005 M aqueous solution of sodium phosphate for 3000 min with a maximum release of TBA and 3,4D of 53.4% and 27.8%, respectively. The release patterns of both anions were fast for the first 400 min, reaching an equilibrium between 2000 and 3000 min. Moreover, Figure 9 shows that the r^2^ value for the pseudo-second-order was the highest among various models and it was closest to 1, suggesting that the release process of TBA and 3,4D into phosphate aqueous solution followed the pseudo-second-order kinetic model.

## 3. Materials and Methods

### 3.1. Synthesis of Zn/Al-NO_3_ LDH

Zn/Al-NO_3_ LDH with Zn to Al molar ratio, R = 2 was prepared as follows. A 250 mL mixture containing 0.1 mol/L Zn(NO_3_) _2_ ⋅ 6H_2_ O and 0.05 mol/L Al(NO_3_) _3_ ⋅ 9H_2_ O was precipitated with 0.5 mol/L NaOH solution under nitrogen atmosphere until the final pH of 7 was reached. The resulting white slurry was aged in an oil bath shaker at 70 °C for 18 h. The resulting precipitate was retrieved via centrifugation, rinsed with deionised water several times, and dried in an oven at 70 °C for 3 days. The dried LDH was powdered using a mortar and pestle.

### 3.2. Synthesis of Zinc-Aluminium-3,4D-TBA (ZADTX)

**Figure 10:**
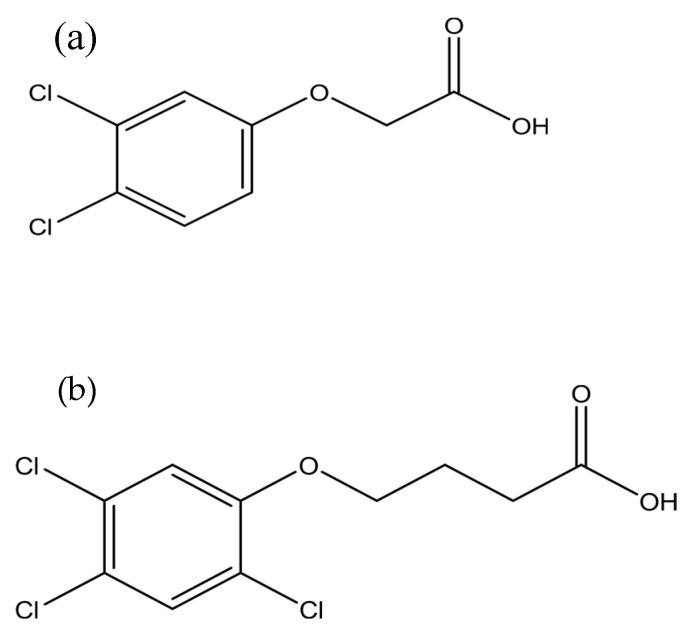
Molecular structures of (**a**) 3,4-dichlorophenoxy-acetic acid (3,4D) and (**b**) 2,4,5-trichlorophenoxybutyric acid (TBA).

In this study, the nanohybrid ZADTX was synthesised via the anion exchange method, in which 0.35 g of LDH was placed into a centrifuge tube containing a 50-mL aqueous solution of 0.1 M 3,4D (98%) and 0.1 M TBA (98%) with molar ratio 1:1 for 3,4D and TBA. Figure 10 shows the molecular structure of 3,4 D and TBA. Reagents of Sigma-Aldrich were used without any further purification, and all solutions were prepared using deionised water. The mixture was stirred for 7 h before being left undisturbed for aging for 18 h at 70 °C in an oil bath shaker. The slurry was then centrifuged at 25 rpm for 5 min at 25 °C, washed with deionised water, and dried in an oven at 70 °C for 72 h. Then, the sample was stored in a sample bottle for further characterisation.

### 3.3. The Physico-Chemical Characterisations and Thermal Analyses

The amounts of 3,4D and TBA anions intercalated into the layered double hydroxide were determined via the UV-visible spectrophotometry with a spectrophotometer (model: Lambda 35, Perkin Elmer, the USA) by first treating the nanohybrid with acid to disintegrate the inorganic layers, fully releasing (un-intercalating) the intercalated anions. Absorbance was monitored at λ_max_ = 219 nm and 222 nm for TBA and 3,4D, respectively. The percentage loading of dual guest nanocomposites was calculated by solving the following simultaneous equations:

at λ222: A1 = Ɛ_tba_ × b × C_tba_ + Ɛ_3,4D_ × b × C_3,4D_ (1)

at λ219: A2 = Ɛ_tba_″ × b × C_tba_ + Ɛ_3,4D_″ × b × C_3,4D_ (2)

where λ222 and λ219 were random wavelength points; A1 and A2 were absorbances for the 10-ppm solution containing both TBA and 3,4D; Ɛ was the absorptivity of each an-ion, C (mg/L) was the concentration of 100 % release of anion, and b was the path length (1 cm).

Meanwhile, The Powder X-ray Diffraction PXRD analyses were conducted at room temperature in the range of 2–60° min^−1^ at 2 °C min^−1^ using a diffractometer (model: XRD-6000, Shimadzu, country) with filtered Cu Kα radiation (λ = 1.5405 Å) at 40kV and 30 mA. Additionally, Fourier-Transform Infrared (FTIR) spectra were recorded in the range of 400–4000 cm^−1^ with a spectrophotometer (model: 1752X, Perkin Elmer, Waltham, MA, USA) using the KBr disc method to make path-lengths small, thus preventing over absorbance by the samples.

Further, thermogravimetric and differential thermogravimetric analysis (TGA-DTG) were performed using a thermogravimetric analyser (model: Mettler Toledo, Switzerland) at a heating rate of 10 °C min^−1^ in the range of 35–1000 °C under nitrogen gas at a flow rate of 50 mL min^−1^. Conversely, the surface property of the material was analysed using a surface area and pore size analyser (model: ASAP2000, Micromeritics, Germany) via the nitrogen gas adsorption-desorption technique at 77 K together with the Brunauer–Emmett–Teller (BET) equation. Samples were degassed at 105 °C for 6 h in an evacuated and heated chamber. Also, the Barrett, Joyner, and Halenda (BJH) procedure was used to calculate pore size distributions from experimental isotherms using the Kelvin model [31].

### 3.4. The Analyses of Release Kinetics

The simultaneous release of 3,4D and TBA from the ZADTX nanohybrid was induced by adding 11.4 mg ZADTX into 100 mL of 0.005 M sodium phosphate aqueous solution. The quantity of the phenoxy herbicide released into the solution was measured at a pre-set time using the UV-visible spectrophotometer at k = 190.0 and 219.0 nm for 3,4D and 222 nm for TBA, respectively. The phosphate anions acted as the incoming exchangeable anion to replace the guests inside the inorganic LDH interlamellar [32]. Data were fitted to kinetics models of the zeroth (3) [33], first-order (4) [34] and pseudo-second-order below (5) [35]:

Ct = kt + c(3)

−log(1 − C) = kt + c(4)

t/C_t_ = 1/k_2_C_eq_^2^ + (1/q_e_)·t (5)

where C_eq_ and C_t_ were the percentage release of the herbicides at equilibrium, t was the time, and c was a constant.

## 4. Conclusions

In this study, a new ZADTX nanohybrid containing two different anions, 3,4D and TBA was synthesised by the ion-exchange method. Both guest herbicides were both intercalated into the LDH, providing well-ordered monophasic nanohybrid content. Controlled and synchronised releases of the two active agents might be performed at different levels, which were regulated by a pseudo-second-order. This finding reveals the possibility of using monophasic nanohybrids for a controlled release of more than one herbicide simultaneously.

## Figures and Tables

**Table 1:** Empirical formula and d-spacing of Zn/Al-NO_3_, and ZADTX nanocomposite.

Sample	Empirical Formula
Zn-Al(NO_3_)-LDH	[Zn_0.67_Al _0.33_(OH)_2_][NO_3_^−^]_0.33_⋅0.56H_2_O
ZAMDTX	[Zn_0.68_Al _0.32_(OH)_2_] [(C_8_H_5_Cl_2_O_3_^−^]_0.16_ [(C_8_H_5_Cl_3_O_3_ ]_0.16_⋅1.88H_2_ O

**Table 2:** Surface properties of LDH and ZAMDTX.

	Surface Properties		
Sample	BET Surface Area (m^2^/g)	BET Average Diameter (Ǻ)	BJH Average Diameter (Ǻ)
LDH	1.12	224.0	124.00
ZAMDTX	2.75	373.02	588.92

## Data Availability

Study did not report any data.exclude.

## Sample Availability

Samples are available from the authors.
